# Veno-venous extracorporeal membrane oxygenation with a bicaval dual-lumen catheter in a SynCardia total artificial heart patient

**DOI:** 10.1186/1749-8090-8-179

**Published:** 2013-08-05

**Authors:** Sotirios Spiliopoulos, Guenes Dogan, Dilek Guersoy, Maria Rosario Serrano, Reiner Koerfer, Gero Tenderich

**Affiliations:** 1 Department for the surgical therapy of end-stage heart failure and mechanical circulatory support, Heart and Vascular Center Duisburg, 47169 Duisburg, Germany

**Keywords:** Circulatory assist devices, Acute respiratory distress syndrome, Extracorporeal membrane oxygenation

## Abstract

We report the case of a 55 years old caucasian male patient with cardiogenic shock due to an extended myocardial infarction who underwent SynCardia Total Artificial Heart implantation and veno-venous extracorporeal membrane oxygenation with a bicaval dual-lumen cannula for the treatment of adult respiratory distress syndrome.

## Background

Refractory cardiogenic schock can be associated with severe respiratory failure requiring extracorporeal membrane oxygenation. We report the first case of Adult respiratory Distress Syndrome in a Total Artificial Heart patient treated by veno-venous extracorporeal membrane oxygenation with a bicaval dual lumen cannula.

## Case presentation

Refractory cardiogenic shock due to an extended myocardial infarction and biventricular heart failure is an established indication for the implantation of a SynCardiaTotal Artificial Heart [1]. In cases of severe respiratory failure, extracorporeal membrane oxygenation may also become necessary [2]. Usually a dual-site veno-venous or veno-arterial cannulation is then performed. We report the first case of adult respiratory distress syndrome in a SynCardia Total Artificial Heart patient which was successfully treated by veno-venous extracorporeal membrane oxygenation with a single-site bicaval dual-lumen catheter placed in the right internal jugular vein.

Angiography of a 55 years old caucasian male patient with an anterior ST segment-elevation myocardial infarction showed thrombosis of the left anterior descending artery and left main artery disease. Despite angioplasty and inotropic therapy the patient continued to decline. Veno-arterial extracorporeal membrane oxygenation became necessary. Radiology showed severe pulmonary congestion and edema due to cardiac decompensation. Additionally, aspiration during endotracheal intubation resulted into pneumonia and severe pulmonary failure causing an adult respiratory distress syndrome with a severe defect in oxygenation and decreased lung compliance. Prior to the procedure high minute ventilation was required to attain normal PaCO2 levels. The veno-arterial ECMO was explanted and implantation of the SynCardia Total Artificial Heart (SynCardia Systems, Inc, Tucson Arizona, USA) was performed as described above [3]. The patient was weaned from extracorporeal circulation and the Total Artificial Heart took over circulation. Due to pre-existent respiratory failure we decided to implement veno-venous extracorporeal membrane oxygenation (Levitronix Centrimag, Switzerland). The circuit was connected to a 19 F bicaval dual-lumen cannula (Avalon Elite®, Maquet Cardiovascular, Germany) placed in the right internal jugular vein as previously described [4]. Correct placement of the cannula in the inferior vena cava and the right atrium was ensured by transesophageal echocardiography. Due to a coagulation disorder and excessive bleeding, the sternum was initially left open and the patient was brought to the ward. Anticoagulation with heparin was instituted 24 hours later with a target-partial thromboplastin time of 60 seconds. Under ECMO support, antibiotics and fluid-conservative management, the adult respiratory distress syndrome gradually resolved. Total duration of ECMO support was 191 hours. ECMO pump flow ranged between 3,0 and 4,0 L/min with TAH cardiac output ranging between 5,1 and 7,7 L/min for the left and 4,2 and 7,3 L/min for the right artificial ventricle. The bicaval dual-lumen cannula was surgically removed. Initially the patient recovered fully and left the ward, but he unfortunately died at postoperative day 192 due to a sepsis related to a driveline-infection.

## Conclusions

Adult respiratory syndrome is a critical condition with a reported in-hospital mortality of 57.9% [5]. Current therapy includes fluid restriction and volume-controlled ventilation with low tidal-volumes and low plateau pressure levels [6]. Extracorporeal membrane oxygenation is known to be associated with a significant survival benefit [7]. However although there have been numerous reports on extracorporeal membrane oxygenation and ARDS in general, the parallel application of an ECMO and the SynCardia Total Artificial Heart for the treatment of severe respiratory failure has been reported once [2]. In our case respiratory failure resulted from cardiogenic shock and aspiration during endotracheal intubation and did not resume despite preoperative veno-arterial ECMO-therapy. Therefore we had to assume a prolonged postoperative duration of ECMO-support. In this context single-site veno-venous cannulation proved to have numerous advantages: it is simple to perform, it minimizes the risk of cannula dislodgement during patient care, reduces the risk of catheter related infections and finally allows early sternum closure. Single-site venous cannulation could be a reasonable alternative to conventional cannulation for ECMO-support in SynCardia Total Artificial Heart patients with an adult respiratory distress syndrome.

## Consent

The patient has given his consent for the case report to be published.

## Competing interests

The authors declare that they have no competing interests.

## Authors’ contributions

SS drafted the manuscript. GD was involved in the drafting of the manuscript. DG was involved in the drafting of the manuscript. MRS revised the manuscript. RK has given final approval of the version to be published. GT has given final approval of the version to be published. All authors read and approved the final manuscript
